# Leveraging new methodologies for public health crisis management

**DOI:** 10.3389/fpubh.2024.1508417

**Published:** 2024-12-20

**Authors:** Hanan Khalil, Joanne Marcucci, Chaojie Liu

**Affiliations:** Department of Public Health, School of Psychology and Public Health, La Trobe University, Melbourne, VIC, Australia

**Keywords:** crisis management, pluralism, system thinking, evidence based medicine, methodologies & tools

## Abstract

Evidence-based medicine is critical in public health emergencies, offering a framework for decision-making and adaptive healthcare responses. By relying on up-to-date and reliable evidence, EBM enables healthcare systems to respond quickly to evolving crises and ensures efficient resource allocation. This perspective presents the importance of evidence-based medicine in public health emergencies, emphasizing the need for rapid decision-making and preparedness. It identifies challenges from the COVID-19 pandemic, including barriers to evidence synthesis, and explores innovative solutions, including methodological pluralism and systems thinking. The findings highlight that evidence-based medicine improves health care systems’ responsiveness to public health crises, supports the efficient resource allocation, and reinforces the need for flexible strategies that adapt to rapidly evolving information. In particular, the practical implications underscore that, in crisis settings, EBM must expand beyond strict evidence hierarchies to include timely, reasonable, and sometimes intuitive expert judgments, ensuring robust and adaptable responses. In conclusion, while EBM enhances healthcare adaptability and decision-making in emergencies, future responses will benefit from incorporating more diverse and flexible approaches to ensure more resilient and effective public health strategies.

## 1 Introduction

Evidence-Based Medicine (EBM) plays a vital role in public health emergencies by providing a structured framework for decision-making, ensuring that actions are informed by the most reliable and up-to-date evidence. Since its inception, EBM enhances the adaptability of healthcare systems, allowing them to quickly respond to evolving situations and adjust strategies as new data emerges. Additionally, EBM is crucial in mitigating misinformation by offering clear, evidence-based guidance that counters false or misleading information (1). This approach also supports the efficient allocation of resources, directing them to where they are most needed based on empirical evidence, ultimately optimizing outcomes and preserving public health during crises (2, 3). The recent COVID-19 pandemic and other global health emergencies have highlighted the need for solutions that extend beyond EBM principles.

The historical evolution of Evidence-Based Medicine (EBM) in crisis settings reflects a gradual shift toward integrating research and empirical evidence into public health decision-making. Initially, EBM emerged as a framework emphasizing rigorous, often experimental methods like randomized controlled trials (RCTs) to inform policies. Over time, and especially during the COVID-19 pandemic, the limitations of traditional EBM methods became evident. Crises like pandemics require rapid, adaptive responses, where high-quality evidence may not always be available due to uncertainties and time constraints (4).

The COVID-19 pandemic underscored the need for EBM to evolve beyond strict evidence hierarchies. In emergencies, standards of evidence had to be expanded to include reasonable, appropriate, and sometimes intuitive judgments made by professionals with domain expertise. This shift acknowledges that traditional methods may not suffice when high-stakes decisions must be made urgently and with limited data. The pandemic highlighted the importance of flexibility in EBM, advocating for a more inclusive approach that incorporates various forms of evidence, including observational data and expert opinion, thus establishing a broader theoretical base for EBM in crisis management (4).

The article aims to discuss the methodological challenges EBM faces in public health emergencies, including issues with evidence synthesis and application. We propose several innovative approaches that leverage EBM, such as methodological pluralism, systems thinking, and adaptive decision-making, to address the complex realities of health crises.

In developing this perspective, we followed a structured approach to ensure clarity and impact. We began by selecting a focused topic and unique viewpoint, then reviewed relevant literature to provide context and support for our stance. The article is organized to present our main arguments logically, integrating supporting evidence while addressing potential counterpoints to enhance credibility. We conclude with key insights and implications, encouraging readers to consider the topic from a fresh angle. This methodology ensures a well-supported and engaging perspective for our audience.

## 2 The importance of EBM in public health emergencies

Rapid decision-making is crucial in healthcare, especially during emergent situations where time-sensitive decisions can significantly impact outcomes. EBM plays a pivotal role by equipping healthcare professionals and policymakers with transparent, reliable, and evidence-based guidance and recommendations (5). By drawing on a robust foundation of existing research and clinical data, EBM allows for the swift implementation of best practices, ensuring that decisions are informed by the latest and most relevant evidence (6). This approach not only improves the quality of care but also enhances the efficiency of healthcare delivery during crises.

In addition to facilitating immediate decision-making, EBM is instrumental in guiding preparedness efforts and the development of pre-established protocols. By integrating evidence into modelling for emergency planning, healthcare systems can anticipate potential challenges and devise strategies to address them proactively (7). Preparedness enables a rapid, coordinated response to emergencies, reducing the likelihood of miscommunication and errors that can occur under pressure. Furthermore, EBM ensures that these protocols are continually updated to reflect new evidence, maintaining their relevance and effectiveness over time.

The application of EBM in rapid decision-making extends beyond immediate responses to include long-term strategies for managing public health emergencies (8). By leveraging existing evidence, policymakers can design interventions that are effective in the short term but also sustainable in the long run. This comprehensive decision-making approach supports resilience in healthcare systems, enabling them to adapt quickly to evolving situations while maintaining a high standard of care. In this way, EBM serves as a critical tool for navigating the complexities of emergency response, ensuring that decisions are grounded in the best available evidence and that outcomes are optimized for the populations served (4).

The dynamic nature of public health emergencies necessitates adaptive strategies that can evolve based on emerging evidence. Unlike static protocols which may quickly become outdated as situations change, adaptive strategies are designed to be flexible, allowing rapid adjustments as new information becomes available (9). This approach ensures that interventions remain relevant and effective throughout the course of an emergency. In a constantly shifting landscape, where new threats can emerge and existing conditions can escalate or subside, the ability to adapt based on the latest evidence is crucial for maintaining the effectiveness of public health responses (10).

“Evidence-based agility” is a key component of this adaptive approach, enabling responders to adjust interventions in real-time to address evolving challenges. By continuously integrating fresh data and research findings, healthcare professionals and policymakers can refine strategies to better meet population needs. This agility not only improves the immediate response to emergencies but also enhances the resilience of public health systems by ensuring they are equipped to handle future crises. In essence, evidence-based agility enables a dynamic, responsive approach to public health, with interventions continuously optimized based on the most current and robust evidence (11).

Moreover, EBM is crucial in combating misinformation during public health emergencies by ensuring that communication strategies are grounded in accurate, science-based information. In crises, the rapid spread of misinformation can lead to confusion, fear, and harmful behaviors that undermine public health efforts. EBM provides a foundation for clear, authoritative messaging that helps to counteract false information and guide the public toward informed, evidence-backed decisions. By promoting transparency and trust through evidence-based communication, EBM not only protects public health outcomes but also empowers individuals and communities to take appropriate actions in response to emerging threats (12).

Additionally, EBM plays a critical role in informing resource allocation decisions during public health emergencies by identifying interventions with the greatest potential impact on population health. By systematically evaluating the effectiveness of different interventions, EBM guides decision-makers in directing limited resources, such as vaccines or medical supplies, to where they can do the most good. This approach is often guided by ethical considerations and decision-making frameworks that prioritize fairness, equity, and overall benefit. During crises, these frameworks help ensure that resources are allocated based not only on scientific evidence but also in a way that upholds ethical principles, such as prioritizing high-risk populations or those most affected by the emergency. This careful balance of evidence and ethics in resource allocation supports more effective and just responses to public health challenges.

## 3 The recent COVID-19 challenges

The recent pandemic, much like other viral outbreaks and public health emergencies, highlighted the need for evidence-making and decision-making processes that could adapt to an evolving situation where evidence and response were produced simultaneously. As the pandemic unfolded, new data emerged rapidly, requiring a dynamic approach to continuously inform public health responses with the latest evidence. This co-production of evidence and response created a complex, iterative process where decisions had to be made in real-time, often with incomplete or emerging information. Policymakers and healthcare professionals were tasked with navigating this uncertain landscape, balancing the need for swift action with the ongoing development of scientific understanding (13).

Despite the extraordinary volume of research generated—over a quarter of a million scientific papers on COVID-19—some fundamental questions about the virus and its management remain contested. For instance, debates persist over the exact mechanisms of virus transmission, the effectiveness of non-pharmaceutical interventions like masks, social distancing, building closures, remote working, and lockdowns, as well as the trade-offs associated with these measures. Additionally, critical questions about making public spaces like schools and hospitals safe, protecting workers and the public while keeping the economy open, and addressing the deep inequalities exacerbated by the pandemic continue to challenge experts. These unresolved issues highlight the difficulties inherent in managing a global health crisis where science is evolving, and where public health strategies must constantly adapt to new evidence while balancing multiple, often conflicting, priorities.

The COVID-19 pandemic presented several challenges in evidence synthesis (14). Key barriers include the fragmentation of primary studies across multiple databases, inefficiencies in the systematic review process, and the duplication of systematic reviews on the same topic, leading to significant research waste. These challenges have made it difficult for decision-makers to obtain clear, actionable information in a timely manner. To address these issues, solutions such as the creation of platforms for sharing individual participant data, improving the efficiency of systematic reviews through computable readable meta-analyses, and establishing rapid review teams are critical steps toward streamlining evidence synthesis. The release of preprints before the peer-review process, combined with selective reporting, highlights the need for clear guidelines on their inclusion in systematic reviews to ensure transparency in the reporting process. Contextual challenges, such as misleading public health announcements and biases in study reporting by political leaders, further complicate decision-making, especially when governing bodies approve treatments without sufficient information to determine their efficacy. Adhering to Evidence-Based Medicine principles and the application of GRADE criteria for grading evidence are essential for informed decision-making. Finally, funding constraints can pose additional challenges, which can be addressed through innovative solutions such as crowdsourcing and leveraging volunteer expertise.

Infectious disease outbreaks often force decision-makers to make rapid choices under conditions of scientific uncertainty, yet the role of evidence in these contexts is not well understood. This article aimed to define the role of scientific evidence in managing infectious disease outbreaks and recommend strategies to overcome barriers to evidence-informed decision-making. Through a scoping review and expert workshop, the study found that decision-makers prioritize expert advice, epidemiological data, and mathematical modelling, but face challenges due to scientific uncertainties that can lead to conflicting interpretations and public criticism (15). The study concludes that the strain on decision-making is not due to a disregard for evidence but rather the lack of clear, timely, and unambiguous evidence. To improve public health responses, it recommends investing in decision-making competencies, building relationships, and promoting transparency. The relationship between science and public health decision-making is underexplored and warrants more attention to ensure effective crisis management (15).

### 3.1 Methodological limitations

Wolkewitz and Puljak (16) highlighted the methodological challenges encountered in COVID-19 research, especially in analyzing data under the urgent demands of the pandemic. They highlighted key statistical issues, including managing time-dependent clinical data, avoiding biases (e.g., selection, immortal time), and developing suitable analysis strategies. The authors emphasized the complexity of clinical endpoints, such as intensive care admission and survival, which require advanced models to accurately interpret. Additionally, the authors discussed the importance of standardized data collection and rapid evidence synthesis, despite the limitations this urgency may impose on methodological rigor. Challenges with data sharing, rapid reporting, and ensuring research quality under tight timelines were also highlighted, underscoring the balance between speed and accuracy in pandemic research (16).

Moreover, several methodological limitations were encountered in studies examining the effects of environmental and socioeconomic variables on COVID-19 spread. A significant limitation is the reliance on ecological designs, which often use aggregate data at regional or national levels rather than individual-level data. This approach introduces biases and limits the ability to draw causal inferences due to unmeasured confounders, particularly around social contact and movement restrictions. Confounding bias is frequently observed, with many studies failing to adjust for critical factors influencing virus transmission, like social behaviors. Measurement errors also arise, because environmental and socioeconomic variables are often inaccurately measured or misclassified over time and across regions, affecting the consistency and reliability of findings. Additionally, the statistical models used in many studies are often inadequate for the complex relationships involved, as they often do not control for non-linearities and spatial or temporal dependencies, which are crucial in the context of pandemic spread dynamics (17).

## 4 Potential innovative solutions to address public health emergencies

There are several potential solutions and new ways to address public health emergencies beyond the use of evidence-based medicine principles as shown in Table 1.

**Table 1:** List of innovative strategies and their application.

Innovative strategy	Application to public health emergencies	Advantages	Disadvantages
Reevaluating the status quo	Focus on upstream determinants of health, such as family structures, workplace environments, housing conditions and socio-economic factors, to develop more effective and equitable public health interventions.	Addresses root causes, potential for equitable impact	May encounter resistance to change
Rapid risk assessment models	Use epidemiological evidence and air travel statistics to predict disease spread, aiding in proactive public health planning and response efforts.	Allows for proactive planning and responses	Data-dependent, may be resource-intensive
Methodological pluralism	Employ diverse research methods and perspectives from various disciplines to enhance the understanding of public health issues and inform more inclusive policymaking and research.	Adaptable, broad, allows for inclusive policymaking	Resource dependent, requires multidisciplinary coordination
Strengthening causal claims	Integrate mechanistic evidence with probabilistic evidence to strengthen the understanding of how interventions lead to specific health outcomes, ensuring broader applicability in real-world settings.	Provides more robust conclusions, applicable across contexts	High methodological rigor required, potential complexity
Systems thinking	Analyze the different elements within a health system and their interactions and influence to lead to more sustainable and responsive interventions.	Holistic and comprehensive view of health systems	High data demands, complexity of integration

### 4.1 Reevaluating the status quo

There is a pressing need to re-evaluate the status quo in public health by integrating knowledge from diverse perspectives, particularly regarding the upstream causes of disease. These causes include family structures, interaction patterns, occupational behaviors, urban density, housing conditions, workplace environments, and the broader social, economic, and cultural contexts that shape health outcomes (18). Additionally, it’s crucial to consider the mechanisms through which interventions might influence these factors, such as shifting attitudes, beliefs, capabilities, and personal resources. By understanding and addressing these underlying determinants of health, we can develop more effective and equitable public health interventions that go beyond immediate clinical solutions to tackle the root causes of disease (19).

During COVID-19, the health system in Quebec reevaluated traditional management structures, catalyzing a shift toward deeper systemic change rather than maintaining previous practices. For example, the pandemic exposed limitations within Quebec’s public health framework, highlighting the need for more robust and flexible responses to health crises. This recognition led to a reconsideration of recent public management approaches and allowed for the exploration of alternative organizational models. By challenging the status quo, Quebec used the pandemic as an opportunity to pursue structural reforms aimed at enhancing the adaptability and resilience of its healthcare system to better address future health emergencies (20).

### 4.2 Rapid risk assessment models

The rapid risk assessment models developed by De Salazar and colleagues integrate epidemiological evidence with air travel data to estimate the number of imported disease cases into various countries (18, 19). The framework employs a series of analyses based on current epidemiological data, including patterns of disease incidence and transmission, alongside detailed air travel statistics. By examining the volume of incoming flights from high-risk areas, the model aims to predict the potential spread of diseases before they reach new locations.

The core of the model involves regression analysis to estimate the number of expected imported cases. This is done by regressing the number of reported imported cases against the relative volume of air travel from the country. The model assumes a linear relationship between travel volume and case importation, suggesting that an increase in travel volume would proportionally increase the number of imported cases. This assumption simplifies the prediction process but may overlook more complex factors affecting disease spread (21).

To quantify the uncertainty in these estimates, the model uses bootstrap sampling to generate 95% prediction intervals. This statistical technique involves resampling the data to create a range of possible outcomes, providing a measure of confidence in the predictions. By incorporating these intervals, the model accounts for variability and offers a more comprehensive assessment of potential risk, aiding in public health planning and response efforts (22).

Another example of using rapid risk assessment during the pandemic is that, during the COVID-19 pandemic, the European Centre for Disease Prevention and Control (ECDC) employed rapid risk assessment to provide timely, targeted updates on the evolving health crisis. These assessments, such as the sixth update released on March 12, 2020, informed European Union and European Economic Area (EU/EEA) countries of key developments, including case numbers, transmission risks, and the capacity of healthcare systems to handle the surge in cases. This assessment emphasized the need for immediate, proactive mitigation measures, including social distancing, isolation of symptomatic individuals, and preventive actions in healthcare facilities to protect vulnerable populations. By offering current, actionable data, the ECDC’s rapid risk assessments helped guide policy decisions to mitigate COVID-19’s impact on public health systems (23).

### 4.3 Methodological pluralism

Methodological pluralism can be defined as an approach in research and epistemology that advocates using multiple methods or strategies to investigate and understand complex phenomena. It has emerged as a critical response to the heavy reliance on biomedical and epidemiological perspectives in guiding public health policy, often at the expense of socio-economic and alternative viewpoints. This criticism highlights a myopic focus that can occur when public health measures are predominantly based on a narrow range of evidence. For instance, the emphasis on “flattening the curve” (24) and the “hammer and the dance” approach (25) exemplifies how a singular focus on epidemic control strategies can overshadow the broader socio-economic impacts and other critical factors.

This approach acknowledges that different methodologies can offer unique insights into complex phenomena, that a single perspective might miss. A systematic review found that, in the context of COVID-19, this means integrating knowledge from various disciplines—epidemiologists for infection rates, social scientists for the burden of caregiving, and aerosol scientists for understanding disease transmission through airborne particles (26). This multidisciplinary approach not only enhanced the understanding of how viruses spread but also provided a more comprehensive view of the public health challenges from health, social, economic, and cultural perspectives. However, methodological pluralism does present challenges. For instance, it can slow down decision-making, especially in crises, and create difficulties in achieving consensus. Furthermore, integrating evidence from different fields can be complicated by varying standards of what counts as credible evidence (25). Despite these challenges, by embracing methodological pluralism, researchers and policymakers can address the full complexity of health crises, incorporating diverse insights to develop more effective and holistic responses.

Moreover, pluralism during the COVID-19 pandemic is highlighted by the integration of interdisciplinary perspectives beyond traditional biomedical approaches. For instance, aerosol scientists contributed critical insights into airborne transmission, challenging the dominant focus on larger respiratory droplets. Using engineering methods like laser-light scattering, aerosol scientists provided strong evidence of airborne transmission, which had previously been underestimated by public health authorities. This interdisciplinary contribution exemplified epistemic pluralism by introducing alternative scientific viewpoints that improved the understanding of virus transmission and influenced public health guidelines (27).

### 4.4 Strengthening causal claims

Strengthening causal claims in scientific research requires the integration of mechanistic evidence, which is inherently explanatory and addresses the critical question of how an effect might be produced (24). Mechanistic evidence delves into the underlying processes or mechanisms that lead to specific outcomes, offering a deeper understanding of causality. By explaining the “how” behind observed effects, mechanistic evidence complements other forms of evidence, such as statistical correlations, providing a more robust foundation for establishing causal relationships.

To build a strong case for causality, it is essential to combine mechanistic evidence with probabilistic evidence from clinical trials and, when appropriate, non-randomized comparative and observational studies. While randomized controlled trials (RCTs) offer valuable probabilistic estimates, such as effect sizes, they are often conducted in controlled environments that may not fully represent real-world circumstances. Mechanistic evidence helps bridge this gap by offering insights that are applicable across various contexts, thereby enhancing the generalizability of findings and ensuring that causal claims remain valid outside the confines of a controlled trial setting.

Mechanistic evidence can be derived from a variety of sources, including well-conducted laboratory and animal studies, modeling and engineering studies, and careful analysis of real-world events. For instance, pre-pandemic studies on the transmission of comparable respiratory viruses provided essential mechanistic insights into how similar pathogens spread, which proved invaluable during the COVID-19 pandemic. By incorporating such diverse forms of evidence, researchers can strengthen causal claims, ensuring that they are not only statistically significant but also grounded in a thorough understanding of the underlying mechanisms (28).

Erqou et al. (29) discussed methods to strengthen causal claims in COVID-19 research by addressing residual confounding in the design of comparative studies. They compared two approaches for selecting control groups to study long-term outcomes of COVID-19 among veterans with heart failure. In the first approach, the control group consisted of veterans without documented COVID-19, regardless of testing status. The second approach only included veterans who had tested negative for SARS-CoV-2 within a similar timeframe and location as the COVID-19-positive group. The second approach demonstrated weaker associations with mortality and hospital admissions, suggesting reduced confounding due to the more rigorous matching criteria (29).

By ensuring both COVID-19 positive and negative groups were tested, the second approach potentially mitigated biases arising from unmeasured differences in health-seeking behavior or socioeconomic factors that might influence testing. This uniform eligibility criterion helped improve covariate balance and reduced the likelihood of including asymptomatic COVID-19 cases in the control group, thus strengthening the causal inference of long-term COVID-19 effects (30).

### 4.5 Systems thinking

Systems thinking is a powerful approach that emphasizes understanding the interconnectedness and interactions among various components within a system. In the context of public health, this approach is particularly valuable as it enables professionals to address complex health issues by considering the multiple factors and relationships that contribute to these challenges. Rather than viewing health issues in isolation, systems thinking encourages a comprehensive analysis of how different elements within a system interact and influence one another, leading to a more holistic understanding of health (30).

Applying a systems thinking approach in public health allows professionals to view health issues as part of a larger system that includes social, economic, environmental, and biological factors. For instance, tackling obesity requires more than just focusing on individual behavior; it involves examining dietary habits, physical activity, food environments, socioeconomic status, and cultural norms. By considering all these interconnected factors, public health interventions can be more effectively designed to address the root causes of obesity, rather than just treating its symptoms (31).

Moreover, systems thinking supports coordination and collaboration among various stakeholders, which is crucial for addressing public health issues. Public health challenges often require collective action from different sectors, including healthcare providers, government agencies, community organizations, and the private sector. Systems thinking fosters a collaborative environment where these diverse stakeholders can work together, share resources, and align their efforts to achieve common health goals. This coordinated approach not only enhances the effectiveness of public health initiatives but also ensures that interventions are sustainable and responsive to the needs of the community.

In the context of the COVID-19 pandemic, systems thinking was applied to address its complexity and evolving nature. By framing the pandemic as a complex adaptive system (CAS), the analysis incorporated both biological and social dynamics, such as the interactions between humans and pathogens. These interactions, which included pathogen mutation and human adaptation behaviors, created emergent and often unpredictable outcomes, further complicating prevention efforts. Systems thinking, using the DSRP (distinctions, systems, relationships, perspectives) model, enabled a structured examination of key components—identifying leverage points and clarifying assumptions underlying different preventive designs. For instance, preventive technologies like contact tracing and physical distancing nudges were assessed through this model to evaluate their effectiveness and potential user impact. This holistic approach highlighted the importance of viewing pandemics not as static problems but as “wicked” issues that demand adaptable, multifaceted strategies (32).

## 5 Practical implications and recommendations

Table 1 outlines innovative strategies for managing public health emergencies, highlighting their applications, advantages, and disadvantages. Reevaluating the status quo emphasizes addressing upstream health determinants to create equitable interventions, although it may face resistance. Rapid risk assessment models facilitate proactive planning based on epidemiological data, although these models rely on extensive data resources. Methodological pluralism promotes an inclusive approach by integrating varied research perspectives, but it requires significant resources and coordination. Strengthening causal claims combines mechanistic and probabilistic evidence to draw more robust conclusions, aiding in real-world application but demanding methodological rigor. Lastly, systems thinking provides a comprehensive analysis of health systems, enhancing sustainability, but posing challenges in data integration. Together, these strategies represent a holistic, data-driven approach to more effectively address complex public health crises.

The lessons learned from the COVID-19 pandemic highlight several key areas where improvements are essential for better preparedness and response to future crises (33). First, there is a recognized need to strengthen crisis preparation, planning, and scenario testing. This requires transparency and public trust. The absence of fully independent Centers for Disease Control and Prevention in some countries has raised concerns. Over the past few decades, public trust in governments has declined (33). The widespread access to social media adds further complexity in gaining public support and ensuring compliance with public health measures (34). Even EBM tools can be hijacked by researchers with conflicts of interest for their own agenda. Therefore, an independent body with access to national data would play a critical role in assessing the validity of various sources of evidence and guiding national response efforts, including better preparedness for future health emergencies. Embracing methodological pluralism—drawing on varied sources of evidence and perspectives—would further support transparency and enhance public trust. Additionally, rapid risk assessment models that integrate epidemiological data with other predictive factors can support timely, evidence-based decision-making in response to emerging threats.

Second, the need for an expert body and a trusted voice on public health is emphasized. The establishment of a panel composed of multidisciplinary experts representing diverse voices from society—not limited to just health professionals but incorporating perspectives from economics, social sciences, and behavioral science. This would enable governments to adopt a system thinking approach during crises. This panel would provide governments with the necessary advice during crises, ensuring that decisions are informed by a broad range of expertise and that a systems approach is adopted. Such a body would enhance public trust in health measures and ensure that policy decisions are guided by the best available evidence from various fields. Reevaluating the status quo by incorporating these broader perspectives will help ensure that policies are not only reactive but also proactive in addressing root causes.

Third, the importance of enhancing public service capability is highlighted. Governments are urged to authorize better collaboration between jurisdictions and strengthen their collective capabilities, particularly in areas such as data management, digital skills, and communication. By fostering stronger collaboration and improving these critical skills, the public service would be better positioned to respond to crises with agility and efficiency, ultimately leading to better outcomes for the population.

Fourth, ensuring equity in access to essential services within and across countries is critical. It is of utmost importance to protect populations at high risk and the most vulnerable during a public health crisis such as the COVID-19 pandemic, as this often determines the final outcomes of public health measures. Public-owned health services have played a pivotal role in the success of country responses to COVID-19, as they are well positioned to ensure equal access to essential services.

Finally, the need to significantly enhance how governments use data is emphasized. Improving the collection, linking, and sharing of real-time data, taking advantage of modern technology while ensuring privacy and security, is crucial for informed decision-making (35, 36). Additionally, building a culture of evaluation and learning is essential. Establishing a politically independent Office of the Evaluator General is proposed to assess the effectiveness of policies, particularly during crises, and provide recommendations for improvement. This approach would ensure that lessons from past experiences are systematically presented and used to inform future policy and practice.

Future longitudinal evaluations of EBM outcomes in crisis contexts are recommended to assess the effectiveness, adaptability, and long-term impact of these approaches. Such evaluations can help identify best practices, refine intervention strategies, and inform policy development to better prepare for and respond to future public health crises. By continuously analyzing outcomes, decision-makers can ensure that EBM-driven strategies remain robust, responsive to new information, and aligned with evolving public health needs. This will support a resilient health system capable of handling complex, multifaceted challenges in an evidence-informed manner.

## 6 Conclusion

Leveraging Evidence-Based Medicine (EBM) for public health crisis management is crucial to ensuring informed decision-making and effective responses to public health emergencies. EBM enables rapid and adaptive strategies while reducing misinformation and optimizing resource allocation. The significant challenges experienced during the recent COVID-19 pandemic further underscore the need for a dynamic approach that incorporates diverse evidence and perspectives. Moving forward, innovative solutions such as methodological pluralism and systems thinking will play a valuable role in addressing the complexities of public health crises.

## Data Availability

The original contributions presented in the study are included in the article/supplementary material, further inquiries can be directed to the corresponding author.
